# Suicidal Thoughts and Behaviors Among Adults Aged ≥18 Years — United States, 2015–2019

**DOI:** 10.15585/mmwr.ss7101a1

**Published:** 2022-01-07

**Authors:** Asha Z. Ivey-Stephenson, Alex E. Crosby, Jennifer M. Hoenig, Shiromani Gyawali, Eunice Park-Lee, Sarra L. Hedden

**Affiliations:** ^1^Division of Injury Prevention, National Center for Injury Prevention and Control, CDC; ^2^Division of Surveillance and Data Collection, Center for Behavioral Health Statistics and Quality, Substance Abuse and Mental Health Services Administration

## Abstract

**Problem/Condition:**

Suicidal thoughts and behaviors are important public health concerns in the United States. In 2019, suicide was the 10th leading cause of death among persons aged ≥18 years (adults); in that year, 45,861 adults died as a result of suicide, and an estimated 381,295 adults visited hospital emergency departments for nonfatal, self-inflicted injuries. Regional- and state-level data on self-inflicted injuries are needed to help localities establish priorities and evaluate the effectiveness of suicide prevention strategies.

**Period Covered:**

2015–2019.

**Description of System:**

The National Survey on Drug Use and Health (NSDUH) is an annual survey of a representative sample of the civilian, noninstitutionalized U.S. population aged ≥12 years. NSDUH collects data on the use of illicit drugs, alcohol, and tobacco; initiation of substance use; substance use disorders and treatment; health care; and mental health. This report summarizes data on responses to questions concerning suicidal thoughts and behaviors contained in the mental health section among sampled persons aged ≥18 years in all 50 states and the District of Columbia. This report summarizes 2015–2019 NSDUH data collected from 254,767 respondents regarding national-, regional-, and state-level prevalence of suicidal thoughts, planning, and attempts by age group, sex, race and ethnicity, region, state, education, marital status, poverty level, and health insurance status.

**Results:**

Prevalence estimates of suicidal thoughts and behaviors varied by sociodemographic factors, region, and state. During 2015–2019, an estimated 10.6 million (annual average) adults in the United States (4.3% of the adult population) reported having had suicidal thoughts during the preceding year. The prevalence of having had suicidal thoughts ranged from 4.0% in the Northeast and South to 4.8% in the West and from 3.3% in New Jersey to 6.9% in Utah. An estimated 3.1 million adults (1.3% of the adult population) had made a suicide plan in the past year. The prevalence of having made suicide plans ranged from 1.0% in the Northeast to 1.4% in the Midwest and West and from 0.8% in Connecticut and New Jersey to 2.4% in Alaska. An estimated 1.4 million adults (0.6% of the adult population) had made a suicide attempt in the past year. The prevalence of suicide attempts ranged from 0.5% in the Northeast to 0.6% in the Midwest, South, and West and from 0.3% in Connecticut to 0.9% in West Virginia. Past-year prevalence of suicidal thoughts, suicide planning, and suicide attempts was higher among females than among males, higher among adults aged 18–39 years than among those aged ≥40 years, higher among noncollege graduates than college graduates, and higher among adults who had never been married than among those who were married, separated, divorced, or widowed. Prevalence was also higher among those living in poverty than among those with a family income at or above the federal poverty threshold and higher among those covered by Medicaid or the Children’s Health Insurance Program than among those with other types of health insurance or no health insurance coverage.

**Interpretation:**

The findings in this report highlight differences in the adult prevalence of suicidal thoughts, plans to attempt suicide, and attempted suicide during the 12 months preceding the survey at the national, regional, and state levels during 2015–2019. Geographic differences in suicidal thoughts and behavior varied by sociodemographic characteristics and might be attributable to sociodemographic composition of the population, selective migration, or the local cultural milieu. These findings underscore the importance of ongoing surveillance to collect locally relevant data on which to base prevention and intervention strategies.

**Public Health Action:**

Understanding the patterns of and risk factors for suicide is essential for designing, implementing, and evaluating public health programs for suicide prevention and policies that reduce morbidity and mortality related to suicidal thoughts and behaviors. State health departments and federal agencies can use the results from this report to assess progress toward achieving national and state health objectives in suicide prevention. Strategies might include identifying and supporting persons at risk, promoting connectedness, and creating protective environments.

## Introduction

Self-directed violence is a major public health issue in the United States (*1*,*2*). It includes a range of behaviors, from nonsuicidal intentional self-harm (i.e., behavior in which the intention is not to kill oneself, as in self-mutilation) to acts of fatal and nonfatal suicidal behavior (*3*). In 2019, suicide was the 10th leading cause of death for U.S. persons aged ≥18 years (adults), resulting in 45,861 deaths (*4*). Suicide is a problem across all age groups, but rates are particularly high among adults (18.0 per 100,000 population) compared with youths aged 10–17 years (4.9 per 100,000 population) (*4*). Apart from a decrease in 2019 rates, suicide rates have risen by approximately 30% since 1999, with rates increasing significantly in most states (*5*). As devastating as suicides are, these deaths represent the smallest proportion of the total public health burden of suicidal behavior. For every adult who died by suicide (*4*), approximately three were hospitalized for nonfatal suicidal behaviors (*6*), nine were seen in an emergency department for suicidal ideation or behavior (*4*), 31 reported having attempted suicide in the past year (*7*), and 234 reported having seriously considered suicide (*7*). In addition, thousands more are affected by the suicides or suicide-related injuries of friends or family members (*8*,*9*). A public health approach and broad array of assessment tools are needed to gain a better understanding of the full magnitude of the problem.

The public health approach to prevention comprises four steps: 1) assessing the magnitude of the problem, 2) identifying the risk and protective factors, 3) developing and evaluating interventions and policies to determine what is successful in preventing the condition or contributing factors, and 4) encouraging widespread adoption of evidence-based programs and policies (*10*). Various assessment tools are used to understand the extent of suicidal thoughts and behaviors and to collect data on deaths and nonfatal injuries in the United States. The National Vital Statistics System (NVSS) compiles demographic and causal data on all deaths filed in the United States. NVSS final mortality data include all fatalities in the United States but provide limited information about the exact nature and circumstances of the injuries (*11*). The National Violent Death Reporting System (NVDRS) provides detailed information about the circumstances surrounding violent deaths, including suicides, drawn from law enforcement reports, coroner and medical examiner reports, and death certificates (*12*). Increases in congressional appropriations allow NVDRS to provide a more comprehensive picture of deaths resulting from suicide; data are being collected for all 50 states, the District of Columbia (DC), and Puerto Rico (*13*). The National Hospital Care Survey (NHCS) and the Health Care and Utilization Project (HCUP) measure inpatient care and hospital use for nonfatal injuries (*14*). Although NHCS and HCUP provide detailed information about the nature of injuries, treatment, and discharge disposition, both are limited because not all states require external cause-of-injury coding, which identifies the intent of the injury on hospital discharge records. Although 47 states and DC provide hospital inpatient data to HCUP, only 36 states and DC provide hospital emergency department data (*15*). The National Electronic Injury Surveillance System–All Injury Program (NEISS-AIP) and NHCS collect data on all types and external causes of nonfatal injuries and poisonings treated in either U.S. hospital emergency departments or hospital outpatient departments (*4*,*14*). Although these systems are nationally representative, they do not have state-level samples. The National Survey on Drug Use and Health (NSDUH) complements data from these other systems. Population surveys such as NSDUH can capture data on injuries that do not result in death or severe injury and that are treated outside the hospital environment in settings (e.g., home) for which vital statistics or hospital-based data are unavailable (*7*).

Population survey data can also supplement information from routine and emergency health care system encounters in addition to complementing data from other systems. Typically, symptom presentation and disease occurrence are captured differently when self-reported rather than when based on a diagnosis by a medical professional. For example, persons might feel more comfortable reporting suicide-related experiences on surveys than they do with their health care providers (*16*). In addition, many persons tend to report fewer health problems to a health care provider than they actually have (*17*,*18*). Anonymous population surveys can be perceived to be less intimidating to a respondent than reporting to a potential authority figure such as a physician or other health care provider (*16*) and thus can often provide more comprehensive data for making population-level comparisons (*19*). Population surveys can provide researchers with a different perspective on the prevalence of suicidal thoughts and behaviors than those provided by death certificates or medical records.

To be effective, population surveys should be made relevant to the populations they monitor. National and state estimates of the prevalence of suicidal thoughts and behaviors can be used to understand the overall public health burden, demonstrate the magnitude of the problem, and establish national and state health priorities (*20*). Nationally representative data also can be used to examine differences in rates among specific groups (e.g., by age, sex, race, and ethnicity) and geographic regions. Large national surveys also allow for aggregating sufficient numbers of particular types of infrequent injuries for identifying patterns and mechanisms. However, national data alone convey an incomplete picture of the public health burden of suicide-related events because considerable variation exists between geographic areas (*21*).

Although a previous report provided much needed state-level data on suicide rates (*5*), state-level prevalence data for suicidal thoughts and behaviors are still lacking, which limits the ability of state prevention specialists to target specific populations on which to focus their primary suicide prevention efforts. Population-based surveys can provide some of this necessary information. This report is a follow-up to a previous report (*22*) and provides more recent state-level data on suicidal thoughts, planning, and attempts among adults as well as prevalence estimates by region, age group, sex, race and ethnicity, and selected characteristics. Gathering data about nonfatal suicidal behavior is integral to prevention efforts because persons who make suicide attempts are one of the highest risk groups for subsequent death from suicide; persons who make suicide attempts are approximately two times more likely to die by suicide than those without a prior history of a suicide attempt (*23*). State health departments and federal agencies can use these state-level data to evaluate progress toward implementing strategies and approaches based on the best available evidence for suicide prevention (*24*) and to assess progress toward achieving national and state health objectives as part of the National Strategy for Suicide Prevention (*2*).

## Methods

This report summarizes national estimates of suicidal thoughts and behaviors including serious thoughts of killing oneself, having a plan, attempting suicide, and suicide attempts requiring medical attention by age group, sex, race and ethnicity, education, marital status, poverty level, health insurance status, and county type (i.e., based on population size). This report also presents regional and state-specific estimates for suicidal thoughts and behaviors by age group, sex, and race and ethnicity. This study was reviewed and approved by the Research Triangle International institutional review board.*

NSDUH is the primary source for statistical information about illicit drug use, alcohol use, substance use disorders, and mental health issues for the civilian, noninstitutionalized U.S. population aged ≥12 years. The survey covers residents of households and persons in noninstitutional group quarters (e.g., shelters, boarding houses, college dormitories, migratory workers’ camps, and halfway houses). Persons with no fixed address (e.g., unhoused persons not in shelters), active-duty military personnel, and residents of institutional group quarters (e.g., jails, nursing homes, mental institutions, and long-term care hospitals) are excluded from the survey. NSDUH is conducted annually by the Substance Abuse and Mental Health Services Administration; data sources have been described in detail previously (*25*–*29*). NSDUH is the only ongoing survey that collects national and state-level data on suicidal thoughts and behaviors among adults. This report analyzes 2015–2019 NSDUH data collected from 254,767 respondents aged ≥18 years.

### Sample Design

NSDUH uses a stratified multistage area probability sample designed to be representative of both the nation and each of the 50 states and DC. Each year, NSDUH has an annual target sample size of 67,500 interviews distributed across three age groups, with 25% allocated to youths aged 12–17 years, 25% to young adults aged 18–25 years, and 50% to adults aged ≥26 years (*25*–*29*).

### Questionnaire

During 2015–2019, all adult NSDUH respondents were asked whether they had thought seriously about trying to kill themselves at any time during the preceding 12 months.^†^ Respondents who reported having had serious thoughts of suicide were then asked whether they had made a plan to kill themselves in the past 12 months and whether they had attempted suicide in the past 12 months. In addition, respondents who reported making a suicide attempt in the past year were asked if they had received medical attention from a doctor or other health care professional because of the attempt. Those who reported having received medical attention were then asked if they had stayed in a hospital overnight or longer after their suicide attempt (*25*–*29*).

### Data Collection and Processing

The NSDUH data collection method involved conducting in-person interviews with a sample of persons residing in the households sampled for the survey. Confidentiality was emphasized in all written and oral communications with prospective respondents. The interview incorporated procedures designed to increase respondents’ cooperation and willingness to report honestly about sensitive issues; audio computer-assisted self-interviewing (ACASI) was used to provide respondents with a private and confidential mode for answering questions about illicit drug use, mental health, and other sensitive behaviors (e.g., suicidal thoughts and suicide attempts). NSDUH interviewers also used computer-assisted personal interviewing (CAPI) to read questions that are less sensitive (e.g., those concerning demographics) to respondents who then entered their answers in a laptop computer. For any given survey year, interview data were compiled in a data file consisting of one record for each completed interview. Some editing and consistency checks were built into ACASI and CAPI during the interview. In addition, more complex edits and consistency checks were applied after the interview was completed during data processing.

### Weighting and Response Rates

The approach to developing and calibrating analysis weights involved developing design-based weights as the product of the inverse of the selection probabilities at each selection stage. Adjustment factors then were applied to account for nonresponse, post-stratify to known population control totals, and to control for extreme weights when necessary. Because this report combines data for 5 years, person-level weights for estimates of annual averages were obtained by dividing the analysis weights by five. Weighted household screening response rates were 79.7% in 2015, 77.9% in 2016, 75.1% in 2017, 73.3% in 2018, and 70.5% in 2019. Weighted interviewing response rates were 69.3% in 2015, 68.4% in 2016, 67.1% in 2017, 66.6% in 2018, and 64.9% in 2019 (*24*–*28*).

### Statistical Analyses

National-, regional-, and state-level annual average prevalence estimates (weighted numbers, percentages, and 95% CIs) were calculated for adults who reported having had suicidal thoughts, made any suicide plans, or attempted suicide in the past 12 months, and were analyzed by selected sociodemographic characteristics: sex, age group (18–39 years, 40–55 years, and ≥56 years), and race and ethnicity (non-Hispanic White, Black, Asian, American Indian or Alaska Native [AI/AN]), Native Hawaiian or Other Pacific Islander [NH/OPI], two or more races, and Hispanic^§^). Weighted estimates were also generated at the national level for adults who had received medical attention and for those who had stayed in a hospital overnight or longer for their suicide attempt. Estimates were examined by selected sociodemographic characteristics: sex, age group, race and ethnicity, educational level (less than high school, high school graduate, some college, college graduate, or more), county type (large metropolitan area, small metropolitan area, and nonmetropolitan area), marital status (never married, married, separated, divorced, or widowed), poverty level (<100%, 100%–199%, or ≥200% of the federal poverty threshold), health insurance (private health insurance, Medicaid or Children’s Health Insurance Program [CHIP], other, or no health insurance), and geographic region^¶^). Definitions of county type are based on U.S. Department of Agriculture classifications: large metropolitan areas have a population of ≥1 million persons, small metropolitan areas have a population of <1 million persons, and nonmetropolitan areas are outside of metropolitan statistical areas (*30*). Chi-square and t-tests were performed to assess statistical significance at a p value of <0.05 of the associations between suicidal thoughts and behaviors (including receiving medical attention for suicide attempt) and sociodemographic characteristics at the bivariate level.

SAS-callable SUDAAN was used to account for the complex sample design and sampling weights. Population subgroups were compared across three or more levels of a categorical variable and conducted log-linear chi-square tests of independence using SUDAAN to first control for type I error inflation resulting from multiple comparisons. If Shah’s Wald F test (transformed from the standard Wald chi-square) indicated overall significant differences, the significance of each pairwise comparison of interest was tested. A p value of <0.05 was considered statistically significant.

Estimates with large sampling errors are considered unreliable and are not reported. The suppression criteria are based on the relative standard error (RSE, defined as the ratio of the standard error over the estimate), actual sample size, and effective sample size for each estimate. According to the suppression criteria, prevalence estimates are suppressed if any of the following occurred: 1)the prevalence estimate is <0.005% or >99.95%, 2) the RSE of the negative natural logarithm of the estimated proportion p (where p is the prevalence divided by 100) is >0.175 if the prevalence is ≤50%, 3) the RSE of the negative of the natural logarithm of (1-p) is >0.175 if the prevalence is >50%, 4) the actual sample size is <100, or 5) the effective sample size (defined as the actual sample size divided by the design effect) is <68. The NSDUH suppression rules have been published previously (*31*). In this report, average annual estimated numbers are reported in thousands. Average annual estimated percentages (prevalence) are rounded to the nearest tenth, and therefore some estimates are shown as <0.1%.

## Results

### Suicidal Thoughts and Behaviors

The prevalence of suicidal thoughts and behaviors is presented by sociodemographic characteristics (Table 1). During 2015–2019, an estimated 10.6 million (annual average) adults in the United States (4.3% of the adult population) reported having had suicidal thoughts in the past year. An estimated 3.1 million adults (1.3% of the adult population) had made suicide plans, and approximately 1.4 million adults (0.6% of the adult population) had attempted suicide in the past year. Females were more likely than males and adults aged 18–39 years were more likely than those aged ≥40 years to have had suicidal thoughts, made plans to kill themselves, or attempted suicide in the past year.

**TABLE 1:** Annual average estimated number* and percentage of adults aged ≥18 years who had suicidal thoughts, made any suicide plans, or attempted suicide during the previous year, by selected demographic characteristics — National Survey on Drug Use and Health, United States, 2015–2019^†^

Characteristic	Suicidal thought^§^		Suicide plan		Suicide attempt	
	No.	% (95% CI)	No.	% (95% CI)	No.	% (95% CI)
Sex						
**Male**	**4,860**	**4.1 (4.0–4.3)**	**1,356**	**1.1 (1.1–1.2)**	**565**	**0.5 (0.4–0.5)**
**Female**	**5,727**	**4.5 (4.4–4.6)**	**1,728**	**1.4 (1.3–1.4)**	**820**	**0.6 (0.6–0.7)**
Age group (yrs)						
**18–39**	**6,515**	**6.9 (6.8–7.1)**	**2,004**	**2.1 (2.1–2.2)**	**968**	**1.0 (1.0–1.1)**
**40–55**	**2,218**	**3.4 (3.2–3.6)**	**655**	**1.0 (0.9–1.1)**	**252**	**0.4 (0.3–0.5)**
**≥56**	**1,854**	**2.2 (2.0–2.3)**	**425**	**0.5 (0.4–0.6)**	**166**	**0.2 (0.1–0.3)**
**Race/Ethnicity**						
White, non-Hispanic	7,169	4.6 (4.4–4.7)	2,014	1.3 (1.2–1.3)	805	0.5 (0.5–0.6)
Black, non-Hispanic	1,081	3.7 (3.5–4.0)	349	1.2 (1.1–1.4)	204	0.7 (0.6–0.8)
Asian, non-Hispanic	400	2.9 (2.5–3.4)	121	0.9 (0.7–1.1)	64	0.5 (0.3–0.6)
AI/AN, non-Hispanic	68	5.0 (4.2–6.0)	26	1.9 (1.4–2.7)	11	0.8 (0.5–1.1)
NH/OPI, non-Hispanic	34	3.8 (2.5–5.8)	11	1.3 (0.5–3.4)	9	0.9 (0.3–3.4)
Two or more races, non-Hispanic	319	7.7 (6.8–8.7)	116	2.8 (2.4–3.3)	51	1.2 (1.0–1.6)
Hispanic^¶^	1,514	3.9 (3.6–4.1)	446	1.1 (1.0–1.3)	243	0.6 (0.5–0.7)
**Education level**						
Less than high school	1,299	4.2 (3.9–4.5)	436	1.4 (1.2–1.6)	292	0.9 (0.8–1.1)
High school graduate**	2,689	4.4 (4.2–4.7)	878	1.5 (1.4–1.6)	435	0.7 (0.6–0.8)
Some college	4,148	5.5 (5.3–5.7)	1,200	1.6 (1.5–1.7)	484	0.6 (0.6–0.7)
College graduate or higher	2,450	3.2 (3.0–3.3)	569	0.7 (0.7–0.8)	174	0.2 (0.2–0.3)
**County type^††^**						
Large metropolitan area^§§^	5,617	4.1 (4.0–4.2)	1,575	1.2 (1.1–1.2)	715	0.5 (0.5–0.6)
Small metropolitan area^¶¶^	3,438	4.7 (4.5–4.9)	1,005	1.4 (1.3–1.5)	449	0.6 (0.6–0.7)
Nonmetropolitan area***	1,532	4.4 (4.2–4.7)	504	1.5 (1.3–1.6)	221	0.6 (0.5–0.7)
**Marital status^†††^**						
Never married	5,440	7.8 (7.6–8.0)	1,662	2.4 (2.3–2.5)	816	1.2 (1.1–1.2)
Married	3,174	2.5 (2.4–2.6)	779	0.6 (0.6–0.7)	271	0.2 (0.2–0.3)
Separated, divorced, or widowed	1,973	4.1 (3.8–4.3)	643	1.3 (1.2–1.5)	298	0.6 (0.5–0.7)
**Poverty level^§§§^**						
<100%	2244	6.6 (6.2–6.9)	763	2.2 (2.1–2.4)	441	1.3 (1.2–1.4)
100%–199%	2427	5.0 (4.8–5.3)	767	1.6 (1.5–1.7)	360	0.7 (0.7–0.8)
≥200%	5827	3.6 (3.5–3.7)	1,527	0.9 (0.9–1.0)	574	0.4 (0.3–0.4)
**Health insurance** ^¶¶¶^						
Private	5,824	3.6 (3.4–3.7)	1,506	0.9 (0.9–1.0)	603	0.4 (0.3–0.4)
Medicaid or CHIP****	2,451	7.0 (6.7–7.3)	874	2.5 (2.3–2.7)	487	1.4 (1.2–1.5)
Other**^††††^**	2,172	3.2 (3.0–3.5)	632	0.9 (0.9–1.0)	248	0.4 (0.3–0.4)
No coverage	1,480	6.1 (5.8–6.4)	470	1.9 (1.7–2.1)	234	1.0 (0.8–1.1)
**Total^§§§§^**	**10,586**	**4.3 (4.2–4.4)**	**3,084**	**1.3 (1.2–1.3)**	**1,385**	**0.6 (0.5–0.6)**

**Abbreviations:** AI/AN = American Indian/Alaska Native; CHIP = Children’s Health Insurance Program; NH/OPI = Native Hawaiian or Other Pacific Islander.

* In thousands.

^†^ Estimates are based only on responses to suicide items in the Mental Health module. Respondents with unknown suicide information were excluded. Only respondents who reported suicide ideation were asked about suicide plans and attempts.

^§^ Respondents who answered “yes” to the question, "At any time in the past 12 months, did you seriously think about trying to kill yourself?" were categorized as having serious thoughts of suicide in the past year.

^¶^ Persons of Hispanic origin can be of any race.

** Includes persons with a general education diploma.

^††^ County type estimates are based on the 2013 Rural-Urban Continuum Codes. https://www.ers.usda.gov/data-products/rural-urban-continuum-codes.aspx

^§§^ Area with a population of ≥1 million persons.

^¶¶^ Area with a population of <1 million persons.

*** Area that is outside of a metropolitan statistical area.

**^†††^** Methodological changes that occurred in 2015 had minimal effects on estimates provided in this report.

^§§§^ Estimates are based on a definition of the poverty level that incorporates information on family income, size, and composition and is calculated as a percentage of the U.S. Census Bureau's poverty thresholds. Respondents aged 18–22 years who were living in a college dormitory were excluded.

^¶¶¶^ Respondents could indicate multiple types of health insurance; thus, these response categories are not mutually exclusive.

**** Persons aged ≤19 years are eligible for this plan.

**^††††^** Defined as having Medicare, CHAMPUS, TRICARE, CHAMPVA, Veterans Administration, military health care, or any other type of health insurance.

^§§§§^ Totals exclude persons with missing or unknown race and ethnicity. Totals might vary due to rounding**.**

Compared with other single racial and ethnic groups, non-Hispanic AI/AN persons had the highest prevalence of having made any suicide plans (Table 1). Non-Hispanic persons of two or more races had higher prevalence rates of having had suicidal thoughts compared with persons of other racial and ethnic groups. During 2015–2019, adults who were college graduates or had more education were less likely to have had suicidal thoughts, made suicide plans, and attempted suicide in the past year than noncollege graduates. Adults with some college education had higher prevalence of having suicidal thoughts, making suicide plans, and attempting suicide than college graduates. During 2015–2019, adults residing in large metropolitan areas were less likely to have made any suicide plans than those in small metropolitan and nonmetropolitan areas. Adults who had never been married had the highest prevalence of past-year suicidal thoughts, plans, and attempts followed by those who were separated, divorced, or widowed, and then those who were married. Adults who were living in poverty (i.e., had a family income of <100% of the federal poverty threshold) had the highest prevalence of past-year suicidal thoughts, plans, and attempts followed by those with family income at 100%–199% above the federal poverty threshold, and then those with family income at 200% or above the federal poverty threshold. Adults with Medicaid or CHIP reported a higher prevalence of having suicidal thoughts, making any suicide plans, and attempting suicide than those with private or other types of health insurance coverage.

During 2015–2019, the estimated prevalence of suicidal thoughts for adults aged ≥18 years ranged from 3.3% in New Jersey to 6.9% in Utah (Table 2). The estimated prevalence rates of adults having made suicide plans in the past year ranged from 0.8% in New Jersey and Connecticut to 2.3% in Alaska. During 2015–2019, approximately 0.6% of adults reported attempting suicide in the past year.

**TABLE 2:** Annual average estimated number* and percentage of adults aged ≥18 years who had suicidal thoughts, made any suicide plans, or attempted suicide during the previous year, by state and geographic region — National Survey on Drug Use and Health, United States, 2015–2019^†^

Region/State	Suicidal thought^§^			Suicide plan			Suicide attempt		
	No.	% (95% CI)	p value (national vs state/region)	No.	% (95% CI)	p value (national vs state/region)	No.	% (95% CI)	p value (national vs state/region)
**Geographic region^¶^**									
Northeast	1,750	4.0 (3.8–4.3)	0.014**	442	1.0 (0.9–1.1)	0**	220	0.5 (0.4–0.6)	0.143
Midwest	2,340	4.5 (4.3–4.8)	0.019**	741	1.4 (1.3–1.6)	0.001**	317	0.6 (0.5–0.7)	0.125
South	3,704	4.0 (3.9–4.2)	0**	1,084	1.2 (1.1–1.3)	0.013**	521	0.6 (0.5–0.6)	0.977
West	2,792	4.8 (4.6–5.1)	0**	818	1.4 (1.3–1.5)	0.003**	326	0.6 (0.5–0.6)	0.960
**State**									
Alabama	146	4.0 (3.3–4.8)	0.350	41	1.1 (0.8–1.6)	0.470	19	0.5 (0.3–0.8)	0.646
Alaska	32	6.1 (5.4–7.0)	0**	12	2.4 (1.9–2.9)	0**	4	0.8 (0.5–1.2)	0.151
Arizona	239	4.5 (3.9–5.3)	0.541	81	1.5 (1.2–1.9)	0.118	40	0.8 (0.5–1.0)	0.131
Arkansas	106	4.8 (3.9–5.8)	0.352	31	1.4 (1.0–1.9)	0.531	15	0.6 (0.4–1.0)	0.569
California	1,259	4.2 (3.9–4.6)	0.679	358	1.2 (1.0–1.4)	0.544	148	0.5 (0.4–0.6)	0.227
Colorado	259	6.1 (5.1–7.3)	0.002**	72	1.7 (1.3–2.2)	0.074	24	0.6 (0.4–0.9)	0.949
Connecticut	100	3.6 (3.0–4.4)	0.059	22	0.8 (0.5–1.2)	0.005**	7	0.3 (0.2–0.4)	0**
Delaware	39	5.3 (4.4–6.3)	0.047**	10	1.4 (1.0–1.9)	0.611	4	0.6 (0.3–1.0)	0.856
District of Columbia	21	3.7 (3.0–4.6)	0.130	6	1.0 (0.7–1.5)	0.228	3	0.6 (0.4–1.0)	0.762
Florida	588	3.6 (3.2–4.1)	0.001**	153	0.9 (0.8–1.1)	0.001**	74	0.5 (0.4–0.6)	0.069
Georgia	296	3.9 (3.3–4.5)	0.152	93	1.2 (0.9–1.6)	0.805	50	0.7 (0.4–1.0)	0.504
Hawaii	46	4.4 (3.5–5.4)	0.910	13	1.3 (0.9–1.8)	0.974	6	0.6 (0.4–0.9)	0.816
Idaho	70	5.6 (4.8–6.5)	0.007**	25	2.0 (1.6–2.4)	0.001**	7	0.5 (0.3–0.8)	0.748
Illinois	351	3.6 (3.2–4.1)	0.004**	115	1.2 (0.9–1.5)	0.644	51	0.5 (0.4–0.7)	0.694
Indiana	275	5.5 (4.7–6.5)	0.008**	98	2.0 (1.5–2.6)	0.008**	43	0.9 (0.6–1.3)	0.074
Iowa	111	4.7 (3.9–5.7)	0.425	42	1.8 (1.3–2.4)	0.053	20	0.9 (0.6–1.3)	0.105
Kansas	112	5.3 (4.4–6.3)	0.061	35	1.6 (1.2–2.2)	0.135	15	0.7 (0.4–1.1)	0.407
Kentucky	171	5.1 (4.3–6.0)	0.071	56	1.7 (1.2–2.2)	0.108	25	0.8 (0.5–1.1)	0.192
Louisiana	153	4.4 (3.8–5.2)	0.739	55	1.6 (1.2–2.1)	0.160	31	0.9 (0.6–1.3)	0.033**
Maine	48	4.5 (3.9–5.3)	0.587	13	1.2 (0.9–1.7)	0.890	4	0.4 (0.3–0.6)	0.059
Maryland	187	4.1 (3.4–4.8)	0.499	47	1.0 (0.7–1.4)	0.176	19	0.4 (0.3–0.7)	0.151
Massachusetts	241	4.5 (3.6–5.6)	0.741	55	1.0 (0.7–1.4)	0.187	27	0.5 (0.3–0.8)	0.578
Michigan	312	4.1 (3.6–4.6)	0.327	86	1.1 (0.9–1.4)	0.261	42	0.5 (0.4–0.7)	0.813
Minnesota	188	4.5 (3.7–5.3)	0.715	55	1.3 (0.9–1.9)	0.811	17	0.4 (0.3–0.7)	0.127
Mississippi	98	4.5 (3.6–5.5)	0.751	34	1.6 (1.1–2.1)	0.233	15	0.7 (0.5–1.0)	0.339
Missouri	189	4.1 (3.4–5.0)	0.597	57	1.2 (0.9–1.7)	0.935	27	0.6 (0.4–1.0)	0.854
Montana	43	5.3 (4.7–6.1)	0.004**	13	1.6 (1.2–2.1)	0.159	4	0.5 (0.4–0.8)	0.857
Nebraska	62	4.4 (3.7–5.3)	0.774	23	1.6 (1.2–2.1)	0.136	7	0.5 (0.3–0.9)	0.753
Nevada	116	5.2 (4.3–6.2)	0.090	43	1.9 (1.3–2.7)	0.060	17	0.8 (0.5–1.2)	0.255
New Hampshire	56	5.2 (4.5–6.1)	0.028**	17	1.6 (1.1–2.3)	0.231	4	0.4 (0.3–0.5)	0.006**
New Jersey	221	3.3 (2.7–3.9)	0.001**	53	0.8 (0.6–1.1)	0**	28	0.4 (0.3–0.7)	0.117
New Mexico	73	4.7 (3.8–5.7)	0.456	17	1.1 (0.8–1.4)	0.248	7	0.4 (0.3–0.6)	0.159
New York	565	3.7 (3.3–4.2)	0.005**	150	1.0 (0.8–1.2)	0.009**	83	0.5 (0.4–0.7)	0.842
North Carolina	329	4.3 (3.7–4.9)	0.842	101	1.3 (1.0–1.7)	0.752	50	0.6 (0.4–0.9)	0.487
North Dakota	25	4.5 (3.8–5.4)	0.638	8	1.5 (1.0.-2.0)	0.397	3	0.6 (0.4–0.8)	0.995
Ohio	480	5.4 (4.9–6.0)	0**	138	1.6 (1.3–1.9)	0.028**	52	0.6 (0.5–0.7)	0.690
Oklahoma	118	4.1 (3.3–5.0)	0.605	38	1.3 (1.0–1.7)	0.723	17	0.6 (0.4–1.0)	0.841
Oregon	182	5.7 (4.9–6.5)	0.001**	50	1.6 (1.2–2.1)	0.149	18	0.6 (0.4–0.8)	0.990
Pennsylvania	448	4.5 (4.0–5.2)	0.453	111	1.1 (0.9–1.4)	0.308	59	0.6 (0.4–0.8)	0.776
Rhode Island	41	4.9 (4.1–5.8)	0.186	12	1.5 (1.0–2.0)	0.426	5	0.6 (0.4–1.0)	0.662
South Carolina	164	4.3 (3.5–5.3)	0.989	56	1.5 (1.0–2.2)	0.478	20	0.5 (0.3–1.0)	0.786
South Dakota	24	3.8 (3.2–4.7)	0.211	8	1.2 (0.9–1.7)	0.874	4	0.6 (0.4–0.9)	0.727
Tennessee	236	4.6 (4.0–5.5)	0.387	69	1.4 (1.0–1.9)	0.674	35	0.7 (0.5–1.0)	0.355
Texas	726	3.6 (3.2–4.0)	0**	196	1.0 (0.8–1.1)	0**	95	0.5 (0.4–0.6)	0.103
Utah	149	6.9 (5.8–8.3)	0**	44	2.0 (1.5–2.7)	0.013**	17	0.8 (0.6–1.1)	0.096
Vermont	31	6.1 (5.2–7.2)	0.001**	8	1.7 (1.2–2.3)	0.156	3	0.5 (0.3–0.9)	0.685
Virginia	258	4.0 (3.5–4.6)	0.331	72	1.1 (0.9–1.5)	0.387	35	0.6 (0.4–0.8)	0.921
Washington	300	5.3 (4.6–6.2)	0.015**	85	1.5 (1.1–2.0)	0.247	31	0.6 (0.3–1.0)	0.939
West Virginia	70	5.0 (4.3–5.8)	0.100	27	1.9 (1.4–2.5)	0.022**	13	0.9 (0.5–1.6)	0.167
Wisconsin	212	4.8 (4.1–5.6)	0.239	75	1.7 (1.3–2.2)	0.062	35	0.8 (0.5–1.2)	0.185
Wyoming	24	5.4 (4.5–6.4)	0.028**	6	1.4 (1.1–1.8)	0.535	3	0.6 (0.4–0.8)	0.849
**Total**	**10,586**	**4.3 (4.2–4.4)**	**NA**	**3,084**	**1.3 (1.2–1.3)**	**NA**	**1,385**	**0.6 (0.5–0.6)**	**NA**

**Abbreviation:** NA = not applicable.

* In thousands.

^†^ Estimates are based only on responses to suicide items in the Mental Health module. Respondents with unknown suicide information were excluded. Only respondents who reported suicide ideation were asked about suicide plans and attempts.

^§^ Respondents who answered “yes” to the question, "At any time in the past 12 months, did you seriously think about trying to kill yourself?" were categorized as having serious thoughts of suicide in the past year.

^¶^ *Northeast*: Connecticut, Maine, Massachusetts, New Hampshire, New Jersey, New York, Pennsylvania, Rhode Island, and Vermont. *Midwest*: Illinois, Indiana, Iowa, Kansas, Michigan, Minnesota, Missouri, Nebraska, North Dakota, Ohio, South Dakota, and Wisconsin*. South*: Alabama, Arkansas, Delaware, District of Columbia, Florida, Georgia, Kentucky, Louisiana, Maryland, Mississippi, North Carolina, Oklahoma, South Carolina, Tennessee, Texas, Virginia, and West Virginia. *West*: Alaska, Arizona, California, Colorado, Hawaii, Idaho, Montana, Nevada, New Mexico, Oregon, Utah, Washington, and Wyoming.

** Statistical significance at the p<0.05 level.

### Suicidal Thoughts

During 2015–2019, approximately 5.7 million adult females and 4.9 million adult males reported having serious thoughts about suicide in the past year; these numbers account for 4.5% of the adult female population and 4.1% of the adult male population in the United States (Table 3). Prevalence of suicidal thoughts among adult females ranged from 4.2% in the South to 4.9% in the Midwest, while the prevalence among adult males ranged from 3.7% in the Northeast to 4.8% in the West. Prevalence of suicidal thoughts among adult females by state ranged from 3.3% in Georgia to 7.1% in Utah and Wyoming, while the prevalence among adult males ranged from 2.5% in New Jersey to 6.8% in Utah (Supplementary Table 1, https://stacks.cdc.gov/view/cdc/111673).

**TABLE 3:** Annual average estimated number* and percentage of adults aged ≥18 years who had suicidal thoughts, plans, and attempts during the previous year, by geographic region and sex — National Survey on Drug Use and Health, United States, 2015-2019^†^

Geographic region^§^	Male		Female		
	No.	% (95% CI)	No.		% (95% CI)
**Suicidal thought**					
Northeast	765	3.7 (3.4–4.0)	985		4.3 (4–4.7)
Midwest	1,037	4.2 (3.9–4.5)	1,302		4.9 (4.6–5.2)
South	1,686	3.8 (3.6–4.1)	2,018		4.2 (4.0–4.4)
West	1,371	4.8 (4.5–5.2)	1,421		4.8 (4.5–5.1)
**Total**	**4,860**	**4.1 (4.0–4.3)**	**5,727**		**4.5 (4.4–4.6)**
**Suicide plan**					
Northeast	175	0.8 (0.7–1)	267		1.2 (1.0–1.4)
Midwest	331	1.3 (1.2–1.5)	410		1.5 (1.4–1.7)
South	477	1.1 (1.0–1.2)	607		1.3 (1.2–1.4)
West	374	1.3 (1.1–1.5)	444		1.5 (1.3–1.7)
**Total**	**1,356**	**1.1 (1.1–1.2)**	**1,728**		**1.4 (1.3–1.4)**
**Suicide attempt**					
Northeast	67	0.3 (0.3–0.4)	153	0.7 (0.5–0.8)	
Midwest	138	0.6 (0.5–0.7)	179	0.7 (0.6–0.8)	
South	222	0.5 (0.4–0.6)	299	0.6 (0.5–0.7)	
West	138	0.5 (0.4–0.6)	188	0.6 (0.5–0.8)	
**Total**	**565**	**0.5 (0.4–0.5)**	**820**	**0.6 (0.6–0.7)**	

* In thousands.

^†^ Estimates based only on responses to suicide items in the Mental Health module. Respondents with unknown suicide information were excluded.

^§^ *Northeast*: Connecticut, Maine, Massachusetts, New Hampshire, New Jersey, New York, Pennsylvania, Rhode Island, and Vermont. *Midwest*: Illinois, Indiana, Iowa, Kansas, Michigan, Minnesota, Missouri, Nebraska, North Dakota, Ohio, South Dakota, and Wisconsin. *South*: Alabama, Arkansas, Delaware, District of Columbia, Florida, Georgia, Kentucky, Louisiana, Maryland, Mississippi, North Carolina, Oklahoma, South Carolina, Tennessee, Texas, Virginia, and West Virginia. *West*: Alaska, Arizona, California, Colorado, Hawaii, Idaho, Montana, Nevada, New Mexico, Oregon, Utah, Washington, and Wyoming.

During 2015–2019, an estimated 6.5 million persons aged 18–39 years in the United States had suicidal thoughts in the past year (6.9% of adults in this age group) (Table 4). Prevalence in this age group ranged from 6.5% in the South to 7.5% in the Midwest. During 2015–2019, 3.4% (2.2 million) of adults aged 40–55 years and 2.2% (1.9 million) of adults aged ≥56 years had suicidal thoughts in the past year, respectively. Prevalence of suicidal thoughts among adults aged 40–55 years ranged from 3.0% in the Northeast to 3.8% in both the Midwest and the West. Among adults aged ≥56 years, prevalence ranged from 2.0% in the Midwest and the South to 2.6% in the West. State prevalence ranged from 5.1% in DC to 10.3% in Utah among those aged 18–39 years, from 1.9% in North Dakota to 6.6% in Utah among adults aged 40–55 years, and from 0.9% in Alabama to 3.6% in Delaware among adults aged ≥56 years (Supplementary Table 2, https://stacks.cdc.gov/view/cdc/111673).

**TABLE 4:** Annual average estimated number* and percentage of adults aged ≥18 years who had suicidal thoughts, plans, and attempts during the previous year, by geographic region and age group — National Survey on Drug Use and Health, United States, 2015–2019^†^

Geographic region^§^	Age group (yrs)					
	18–39		40–55		≥56	
	No.	% (95% CI)	No.	% (95% CI)	No.	% (95% CI)
**Suicidal thought**						
Northeast	1,073	6.7 (6.3–7)	350	3.0 (2.6–3.5)	328	2.1 (1.7–2.5)
Midwest	1,453	7.5 (7.2–7.8)	509	3.8 (3.4–4.2)	378	2.0 (1.7–2.4)
South	2,272	6.5 (6.2–6.8)	782	3.1 (2.8–3.5)	650	2.0 (1.8–2.3)
West	1,717	7.4 (7–7.8)	577	3.8 (3.4–4.2)	499	2.6 (2.0.2–3)
**Total **	**6,515**	**6.9 (6.8–7.1)**	**2,218**	**3.4 (3.2–3.6)**	**1,854**	**2.2 (2.0–2.3)**
**Suicide plan**						
Northeast	295	1.8 (1.7–2.0)	79	0.7 (0.5–0.9)	68	0.4 (0.3–0.7)
Midwest	478	2.5 (2.3–2.7)	166	1.2 (1.0–1.5)	97	0.5 (0.4–0.7)
South	695	2.0 (1.8–2.1)	246	1.0 (0.8–1.2)	143	0.4 (0.3–0.6)
West	536	2.3 (2.1–2.5)	165	1.1 (0.9–1.3)	117	0.6 (0.4–0.8)
**Total**	**2,004**	**2.1 (2.1–2.2)**	**655**	**1.0 (0.9–1.1)**	**425**	**0.5 (0.4–0.6)**
**Suicide attempt**						
Northeast	166	1.0 (0.9–1.2)	27	0.2 (0.1–0.5)	27	0.2 (0.1–0.3)
Midwest	216	1.1 (1.0–1.2)	63	0.5 (0.3–0.6)	39	0.2 (0.1–0.4)
South	350	1.0 (0.9–1.1)	111	0.4 (0.3–0.6)	60	0.2 (0.1–0.3)
West	236	1.0 (0.9–1.2)	51	0.3 (0.2–0.5)	40	0.2 (0.1–0.4)
**Total**	**968**	**1.0 (1.0–1.1)**	**252**	**0.4 (0.3–0.5)**	**166**	**0.2 (0.1–0.3)**

* In thousands.

^†^ Estimates based only on responses to suicide items in the Mental Health module. Respondents with unknown suicide information were excluded.

^§^ *Northeast*: Connecticut, Maine, Massachusetts, New Hampshire, New Jersey, New York, Pennsylvania, Rhode Island, and Vermont. *Midwest*: Illinois, Indiana, Iowa, Kansas, Michigan, Minnesota, Missouri, Nebraska, North Dakota, Ohio, South Dakota, and Wisconsin. *South*: Alabama, Arkansas, Delaware, District of Columbia, Florida, Georgia, Kentucky, Louisiana, Maryland, Mississippi, North Carolina, Oklahoma, South Carolina, Tennessee, Texas, Virginia, and West Virginia. *West*: Alaska, Arizona, California, Colorado, Hawaii, Idaho, Montana, Nevada, New Mexico, Oregon, Utah, Washington, and Wyoming.

During 2015–2019, an estimated 7.2 million non-Hispanic White adults in the United States (4.6% of the non-Hispanic White adult population) reported having had suicidal thoughts in the past year (Table 5). Among non-Hispanic White adults, prevalence ranged from 4.1% in the Northeast to 5.3% in the West. During that same period, an estimated 1.1 million non-Hispanic Black adults (3.7% of the non-Hispanic Black adult population) reported having had serious thoughts about suicide in the past year. Among non-Hispanic Black adults, estimated prevalence for suicidal thoughts ranged from 3.5% in the South to 4.7% in the West. During 2015–2019, an estimated 400,000 non-Hispanic Asian persons in the United States (2.9% of the non-Hispanic Asian adult population) had suicidal thoughts in the past year. Among non-Hispanic Asian adults, prevalence ranged from 1.6% in the Northeast to 3.8% in the Midwest. During that same period, an estimated 68,000 non-Hispanic AI/AN adults (5.0% of the non-Hispanic AI/AN adult population) reported having had serious thoughts about suicide in the past year. Among non-Hispanic AI/AN adults, prevalence ranged from 3.8% in the Northeast to 7.7% in the Midwest. During 2015–2019, an estimated 1.5 million Hispanic adults (3.9% of the Hispanic adult population) reported having serious thoughts about suicide in the past year. Among Hispanic adults, prevalence ranged from 3.2% in the South to 4.6% in the Northeast.

**TABLE 5:** Annual average estimated number* and percentage of adults aged ≥18 years who had suicidal thoughts, plans, and attempts during the previous year, by geographic region and race/ethnicity — National Survey on Drug Use and Health, United States, 2015–2019^†^

Geographic region^§^	Race/Ethnicity									
	White, non-Hispanic		Black, non-Hispanic		Asian, non-Hispanic		AI/AN, non-Hispanic		Hispanic	
	No.	% (95% CI)	No.	% (95% CI)	No.	% (95% CI)	No.	% (95% CI)	No.	% (95% CI)
**Suicidal thought**										
Northeast	1,228	4.1 (3.8–4.5)	169	3.6 (2.9–4.5)	44	1.6 (1.2–2.0)	2	3.8 (1.9–7.4)	261	4.6 (3.9–5.5)
Midwest	1,846	4.6 (4.3–4.8)	212	4.3 (3.7–5.0)	62	3.8 (2.9–4.9)	18	7.7 (5.7–10.3)	140	4.1 (3.4–4.9)
South	2,428	4.4 (4.1–4.6)	583	3.5 (3.2–3.8)	93	3.0 (2.3–3.9)	21	4.5 (3.0–6.7)	477	3.2 (2.9–3.6)
West	1,667	5.3 (5.0–5.7)	118	4.7 (3.7–5.9)	201	3.3 (2.6–4.1)	27	4.5 (3.4–5.9)	637	4.1 (3.7–4.6)
**Total**	**7,169**	**4.6 (4.4–4.7)**	**1,081**	**3.7 (3.5–4.0)**	**400**	**2.9 (2.5–3.4)**	**68**	**5.0 (4.2–6.0)**	**1,514**	**3.9 (3.6–4.1)**
**Suicide plan**										
Northeast	284	1.0 (0.8–1.1)	45	1.0 (0.7–1.3)	21	0.7 (0.5–1.1)	1	1.4 (0.4–4.3)	74	1.3 (0.9–2.0)
Midwest	562	1.4 (1.3–1.5)	68	1.4 (1.0–1.9)	25	1.5 (1.0–2.4)	6	2.6 (0.9–7.0)	55	1.6 (1.2–2.3)
South	713	1.3 (1.2–1.4)	196	1.2 (1.0–1.3)	16	0.5 (0.3–1.0)	10	2.1 (1.2–3.6)	108	0.7 (0.6–0.9)
West	455	1.5 (1.3–1.6)	40	1.6 (1.0–2.5)	59	1.0 (0.6–1.5)	10	1.6 (1.1–2.4)	208	1.3 (1.2–1.6)
**Total**	**2,014**	**1.3 (1.2–1.3)**	**349**	**1.2 (1.1–1.4)**	**121**	**0.9 (0.7–1.1)**	**26**	**1.9 (1.4–2.7)**	**446**	**1.1 (1.0–1.3)**
**Suicide attempt**										
Northeast	122	0.4 (0.3–0.5)	28	0.6 (0.4–0.9)	13	0.5 (0.3–0.8)	0	0.6 (0.1–2.7)	47	0.8 (0.5–1.4)
Midwest	233	0.6 (0.5–0.7)	37	0.8 (0.5–1.1)	9	0.5 (0.3–1.1)	2	0.9 (0.5–1.8)	25	0.7 (0.5–1.1)
South	302	0.5 (0.5–0.6)	118	0.7 (0.6–0.8)	14	0.5 (0.2–0.9)	3	0.7 (0.3–1.5)	63	0.4 (0.3–0.6)
West	148	0.5 (0.4–0.6)	21	0.8 (0.5–1.3)	27	0.4 (0.2–0.9)	5	0.8 (0.5–1.4)	107	0.7 (0.6–0.9)
**Total**	**805**	**0.5 (0.5–0.6)**	**204**	**0.7 (0.6–0.8)**	**64**	**0.5 (0.3–0.6)**	**11**	**0.8 (0.5–1.1)**	**243**	**0.6 (0.5–0.7)**

**Abbreviation**: AI/AN = American Indian or Alaska Native.

* In thousands.

^†^ Estimates based only on responses to suicide items in the Mental Health module. Respondents with unknown suicide information were excluded.

^§^ *Northeast*: Connecticut, Maine, Massachusetts, New Hampshire, New Jersey, New York, Pennsylvania, Rhode Island, and Vermont. *Midwest*: Illinois, Indiana, Iowa, Kansas, Michigan, Minnesota, Missouri, Nebraska, North Dakota, Ohio, South Dakota, and Wisconsin. *South*: Alabama, Arkansas, Delaware, District of Columbia, Florida, Georgia, Kentucky, Louisiana, Maryland, Mississippi, North Carolina, Oklahoma, South Carolina, Tennessee, Texas, Virginia, and West Virginia. *West*: Alaska, Arizona, California, Colorado, Hawaii, Idaho, Montana, Nevada, New Mexico, Oregon, Utah, Washington, and Wyoming.

Among non-Hispanic White adults, state prevalence ranged from 3.2% in Connecticut to 6.5% in Utah (Supplementary Table 3, https://stacks.cdc.gov/view/cdc/111673). Among states with reliable estimates for non-Hispanic Black adults, prevalence ranged from 2.2% in Arizona to 10.2% in Nebraska; for non-Hispanic Asian adults, prevalence ranged from 1.4% in New Jersey and New York to 5.8% in Nevada. Prevalence for non-Hispanic AI/AN adults ranged from 1.4% in North Carolina to 10.6% in Montana and for Hispanic adults, prevalence ranged from 1.5% in South Dakota to 10.2% in Alaska.

During 2015–2019, approximately 1.3 million adults with less than a high school education reported having had suicidal thoughts in the past year (4.2% of the adult population with less than a high school education) (Table 1). An estimated 2.5 million college graduates reported having had serious thoughts about suicide, representing 3.2% of the adult college graduate population. During 2015–2019, 7.8% (5.4 million) of adults who had never been married had suicidal thoughts in the past year. An estimated 3.2 million adults who were married reported having serious thoughts about suicide in the past year (2.5% of the married adult population). An estimated 2.2 million adults who were living in poverty (i.e., family income of less than 100% of the federal poverty threshold) reported having suicidal thoughts in the past year; this number accounts for 6.6% of the adult population living in poverty. During 2015–2019, 7.0% (2.5 million) of adults covered by Medicaid or CHIP and 6.1% (1.5 million) of adults without health insurance reported having serious thoughts about suicide in the past year, respectively. Approximately, 4.1% (5.6 million) of adults residing in large metropolitan areas had suicidal thoughts in the past year, while 4.4% (1.5 million) of adults residing in non-metropolitan areas reported suicidal thoughts in the past year.

### Suicide Plans

During 2015–2019, an estimated 1.4 million adult males and 1.7 million adult females reported making any suicide plans in the past year, representing 1.1% of adult males and 1.4% of adult females in the United States, respectively (Table 3). Among adult males, prevalence ranged from 0.8% in the Northeast to 1.3% in the Midwest and the West. Prevalence for adult females ranged from 1.2% in the Northeast to 1.5% in the Midwest and the West. An estimated 2.0 million adults aged 18–39 years (2.1% of the population in this age group) made suicide plans. In this age group, prevalence ranged from 1.8% in the Northeast to 2.5% in the Midwest (Table 4). Approximately 1.0% (655,000) of adults aged 40–55 years and 0.5% (425,000) of adults aged ≥56 years made any suicide plans in the past year, respectively. Prevalence of making suicide plans among adults aged 40–55 years ranged from 0.7% in the Northeast to 1.2% in the Midwest. Among adults aged ≥56 years, prevalence estimates ranged from 0.4% in the Northeast and the South to 0.6% in the West.

Among adult males, state prevalence ranged from 0.4% in New Jersey to 2.6% in Alaska whereas prevalence for adult females ranged from 0.8% in Connecticut to 2.6% in Idaho (Supplementary Table 4, https://stacks.cdc.gov/view/cdc/111673). Among adults aged 18–39 years, state prevalence ranged from 1.2% in Connecticut to 4.3% in Alaska; among adults aged 40–55 years, prevalence ranged from 0.4% in New Jersey to 2.4% in Louisiana; and among states with stable estimates for adults aged ≥56 years, prevalence ranged from 0.1% in Maine, Michigan, Missouri, and Tennessee to 1.6% in New Hampshire (Supplementary Table 5, https://stacks.cdc.gov/view/cdc/111673).

During 2015–2019, an estimated 2.0 million non-Hispanic White adults in the United States (1.3% of the non-Hispanic White adult population) made any suicide plans in the past year. Among non-Hispanic White adults, prevalence ranged from 1.0% in the Northeast to 1.5% in the West (Table 5). During the same period, an estimated 349,000 non-Hispanic Black adults (1.2% of the non-Hispanic Black adult population) made suicide plans in the past year. Among non-Hispanic Black adults, prevalence estimates ranged from 1.0% in the Northeast to 1.6% in the West. During 2015–2019, an estimated annual average of 121,000 non-Hispanic Asian adults in the United States (0.9% of the non-Hispanic Asian adult population) made suicide plans in the past year. Among non-Hispanic Asian adults, prevalence estimates ranged from 0.5% in the South to 1.5% in the Midwest. During that same period, an estimated 26,000 non-Hispanic AI/AN adults (1.9% of the non-Hispanic AI/AN adult population) reported having made suicide plans in the past year. Among non-Hispanic AI/AN adults, prevalence estimates ranged from 1.4% in the Northeast to 2.6% in the Midwest. During that same period, an estimated annual average of 446,000 Hispanic adults (1.1% of the Hispanic adult population) reported having made suicide plans in the past year. Among Hispanic adults, prevalence estimates ranged from 0.7% in the South to 1.6% in the Midwest. State prevalence among non-Hispanic White adults ranged from 0.5% in DC to 2.0% in Colorado and West Virginia (Supplementary Table 6, https://stacks.cdc.gov/view/cdc/111673). Among states with reliable estimates for non-Hispanic Black adults, prevalence ranged from 0.7% in New York and South Carolina to 4.2% in Nebraska; for non-Hispanic Asian adults, prevalence ranged from 0.1% in Maryland to 2.6% in Alaska. Prevalence for non-Hispanic AI/AN adults ranged from 0.8% in Arizona and New Mexico to 4.1% in Alaska and for Hispanic adults, prevalence ranged from 0.2% in Louisiana to 5.4% in Alaska.

During 2015–2019, an estimated 436,000 adults with less than a high school education made suicide plans in the past year (1.4% of the adult population with less than a high school education) (Table 1). An estimated 569,000 college graduates reported having made suicide plans, representing 0.7% of the adult college graduate population. During 2015–2019, 2.4% (1.7 million) of adults who had never been married made suicide plans in the past year. An estimated 779,000 adults who were married reported having made suicide plans the past year (0.6% of the married adult population). During 2015–2019, an estimated 763,000 adults who were living in poverty (i.e., family income of <100% of the federal poverty threshold) reported having made suicide plans in the past year; this number accounts for 2.2% of the adult population living in poverty. An estimated 1.5 million adults who were living at ≥200% of the poverty threshold reported having made suicide plans in the past year; this number accounts for 0.9% of that population. During 2015–2019, 2.5% (874,000) of adults covered by Medicaid or CHIP and 1.9% (470,000) of adults without health insurance reported having made suicide plans in the past year, respectively. Approximately, 1.2% (1.6 million) of adults residing in large metropolitan areas reported making suicide plans in the past year, while an estimated 1.5% (504,000) of adults residing in non-metropolitan areas reported suicidal plans in the past year.

### Suicide Attempts

During 2015–2019, an estimated 565,000 adult males and 820,000 adult females reported making a suicide attempt in the past year; these numbers account for 0.5% of adult males and 0.6% of adult females, respectively (Table 3). Prevalence among adult males ranged from 0.3% in the Northeast to 0.6% in the Midwest. Among adult females, prevalence ranged from 0.6% in the South and West to 0.7% in the Northeast and Midwest. Prevalence among adult males by state ranged from 0.2% in New Jersey to 0.9% in Alaska and Kentucky. Among adult females, state prevalence ranged from 0.2% in Connecticut to 1.1% in Louisiana (Supplementary Table 7, https://stacks.cdc.gov/view/cdc/111673).

During 2015–2019, an estimated 968,000 adults aged 18–39 years (1.0% of the population in this age group) attempted suicide in the past year. In this age group, prevalence was similar 1.0% in the Northeast, South, and West to 1.1% in the Midwest (Table 4). Approximately 0.4% (252,000) of adults aged 40–55 years and 0.2% (166,000) of adults aged ≥56 years made a suicide attempt in the past year, respectively. Among regions, prevalence among adults aged 40–55 years ranged from 0.2% in the Northeast to 0.5% in the Midwest. Among adults aged ≥56 years, prevalence of past-year suicide attempt was similar across regions an estimated 0.2%. State prevalence ranged from 0.5% in Washington to 1.7% in Indiana among adults aged 18–39 years (Supplementary Table 8, https://stacks.cdc.gov/view/cdc/111673). Prevalence among adults aged 40–55 years ranged from <0.1% in Maine, New Hampshire, and Rhode Island to 0.8% in Arkansas, DC, and Michigan. Adults aged ≥56 years had prevalence of past-year suicide attempt that ranged from <0.1% in Michigan to 0.7% in Washington.

During 2015–2019, an estimated 805,000 non-Hispanic White adults in the United States (0.5% of the non-Hispanic White adult population) made a suicide attempt in the past year. Among non-Hispanic White adults, prevalence ranged from 0.4% in the Northeast to 0.6% in the Midwest (Table 5). During the same period, approximately 204,000 non-Hispanic Black adults (0.7% of the non-Hispanic Black adult population) attempted suicide in the past year. Among regions, estimates for non-Hispanic Black adults ranged from 0.6% in the Northeast to 0.8% in the Midwest and West. During 2015–2019, an estimated annual average of 64,000 non-Hispanic Asian in the United States (0.5% of the non-Hispanic Asian adult population) attempted suicide in the past year. Among regions, the estimated prevalence for non-Hispanic Asian adults ranged from 0.4% in the West to 0.5% in the Midwest, Northeast, and South. During that same period, an estimated 11,000 non-Hispanic AI/AN adults (0.8% of the non-Hispanic AI/AN adult population) reported attempting suicide in the past year. Among regions, the estimated prevalence for non-Hispanic AI/AN adults ranged from 0.6% in the Northeast to 0.9% in the Midwest. During that same period, an estimated annual average of 243,000 Hispanic adults (0.6% of the Hispanic adult population) reported attempting suicide in the past year. Among regions, the estimated prevalence for Hispanic adults ranged from 0.4% in the South to 0.8% in the Northeast. Among non-Hispanic White adults, state prevalence ranged from 0.1% in DC to 1.0% in West Virginia (Supplementary Table 9, https://stacks.cdc.gov/view/cdc/111673). Prevalence for non-Hispanic Black adults ranged from 0.2% in Rhode Island and West Virginia to 1.8% in Nevada; prevalence for non-Hispanic Asian adults ranged from 0.1% in Oregon to 1.0% in DC. Among non-Hispanic AI/AN adults, estimated prevalence ranged from 0.2% in North Carolina to 2.7% in Montana; estimated prevalence among Hispanic adults ranged from 0.1% in Oklahoma and Washington to 1.8% in Alaska.

During 2015–2019, an estimated 292,000 adults with less than a high school education made at least one suicide attempt in the past year (0.9% of the adult population with less than a high school education) (Table 1). An estimated 174,000 persons with a college degree or more reported having made suicide attempts, representing 0.2% of the adult college graduate population. During 2015–2019, 816,000 (1.2%) adults who had never been married reported making at least one suicide attempt in the past year. An estimated 271,000 adults who were married reported having made at least one suicide attempt the past year (0.2% of the married adult population). During 2015–2019, an estimated 441,000 adults who were living in poverty (i.e., family income of less than 100% of the federal poverty threshold) reported having made a suicide attempt in the past year; this number accounts for 1.3% of the adult population living in poverty. During 2015–2019, 487,000 (1.4%) adults covered by Medicaid or CHIP and 234,000 (1.0%) adults without health insurance reported having made a suicide attempt in the past year, respectively, compared with 603,000 (0.4%) of those with private health insurance. Approximately 715,000 (0.5%) adults residing in large metropolitan areas reported making suicide plans in the past year compared with 221,000 (0.6%) living in nonmetropolitan areas.

### Suicidal Attempts that Required Medical Attention or Hospitalization

During 2015–2019, approximately 741,000 adults received medical attention from a doctor or other health professional in the past year for a suicide attempt, accounting for 53.5% of those who reported a suicide attempt (Table 6). Of the adults who reported a suicide attempt in the past year, 516,000 said they stayed in a hospital overnight or longer (37.3%). Females had a slightly higher percentage of reporting a need for medical attention after a suicide attempt, but males reported a slightly higher percentage that needed hospitalization (neither difference was statistically significant). Adults aged 18–39 years reported a higher prevalence of requiring medical attention and hospitalization for suicide attempts compared with those aged 40–55 years. The sample among adults aged ≥56 years was unstable. Hispanic adults were less likely to report having received medical attention than non-Hispanic White adults; no other differences were noted. During 2015–2019, adults who were never married were less likely to have received medical attention or hospitalization for suicidal attempts than those who were married or those who were separated, divorced, or widowed. Adults without health insurance coverage were less likely to have received medical attention for suicidal attempts than those with health insurance. Prevalence of having received medical attention or hospitalization suicide attempts did not differ by educational attainment, county type, poverty threshold/income level, or geographic region.

**TABLE 6:** Annual average estimated number* and percentage of adults aged ≥18 years who attempted suicide requiring medical attention or hospitalization during the previous year, by selected demographic characteristics — National Survey on Drug Use and Health, United States, 2015–2019^†^

Characteristic	Medical attention		Hospitalization	
	No.	% (95% CI)	No.	% (95% CI)
**Sex**				
Male	299	52.9 (48.0–57.8)	215	38.1 (33.4–43.1)
Female	442	53.9 (50.0–57.8)	301	36.7 (32.8–40.9)
**Age group (yrs) **				
18–39	453	46.8 (43.9–49.8)	299	30.9 (28.3–33.7)
40–55	178	70.6 (62.6–77.4)	133	53.0 (44.7–61.1)
≥56	—^§^	—	—	—
**Race/Ethnicity**				
White, non-Hispanic	458	56.9 (52.9–60.7)	312	38.7 (34.9–42.8)
Black, non-Hispanic	111	54.6 (47.2–61.8)	82	40.2 (33.0–47.9)
Asian, non-Hispanic	—	—	—	—
AI/AN, non-Hispanic	—	—	—	—
NH/OPI, non-Hispanic	—	—	—	—
Two or more races, non-Hispanic	—	—	—	—
Hispanic^¶^	106	43.8 (36.2–51.7)	82	33.8 (26.5–42.1)
**Education level**				
Less than high school	171	58.7 (51.9–65.1)	135	46.1 (39.1–53.3)
High school graduate**	209	48.1 (42.7–53.6)	132	30.5 (25.9–35.5)
Some college	254	52.6 (47.9–57.3)	170	35.2 (30.7–40.0)
College graduate or higher	106	60.9 (50.3–70.5)	79	45.2 (34.9–55.9)
**County type^††^**				
Large metropolitan^§§^	370	51.8 (47.1–56.6)	271	38.0 (33.3–42.9)
Small metropolitan^¶¶^	257	57.3 (52.4–62.2)	176	39.3 (34.3–44.6)
Nonmetropolitan***	113	51.2 (44.0–58.3)	69	31.0 (25.3–37.4)
**Marital status^†††^**				
Never married	375	46.0 (42.8–49.3)	255	31.3 (28.3–34.4)
Married	171	63.2 (54.9–70.7)	115	42.6 (34.5–51.1)
Separated, divorced, or widowed	195	65.2 (57.0–72.6)	146	49.0 (40.5–57.5)
**Poverty level^§§§^**				
<100%	247	56.2 (50.6–61.6)	186	42.3 (36.8–48.1)
100%–199%	181	50.2 (44.2–56.2)	104	28.9 (24.1–34.2)
≥200%	309	53.9 (49.0–58.8)	223	38.9 (34.0–44.1)
**Health insurance**^¶¶¶^				
Private	329	54.6 (50.0–59.1)	221	36.7 (32.2–41.4)
Medicaid or CHIP****	289	59.3 (53.6–64.7)	216	44.3 (38.8–50.0)
Other**^††††^**	149	60 (50.8–68.5)	109	44.0 (35.2–53.1)
No coverage	94	40.2 (33.6–47.1)	62	26.4 (20.7–33.0)
**Geographic region^§§§§^**				
Northeast	118	53.6 (46.3–60.7)	88	40.0 (32.7–47.9)
Midwest	182	57.3 (51.4–62.9)	128	40.4 (34.6–46.5)
South	272	52.2 (47.1–57.3)	183	35.1 (30.3–40.2)
West	169	51.9 (44.9–58.7)	117	36.0 (29.5–43.0)
**Total** ^¶¶¶¶^	**741**	**53.5 (50.3–56.7)**	**516**	**37.3 (34.2–40.5)**

**Abbreviations**: AI/AN = American Indian or Alaska Native; CHIP = Children’s Health Insurance Program; NH/OPI = Native Hawaiian or Other Pacific Islander; RSE = relative standard error.

* In thousands.

^†^ Estimates based only on responses to suicide items in the Mental Health module. Respondents with unknown suicide information were excluded.

^§^ Low precision; no estimate reported. According to the suppression criteria, prevalence estimates are suppressed if any of the following occurred: 1) the prevalence estimate is <0.005% or >99.95%, 2) the RSE of the negative natural logarithm of the estimated proportion p (where p is the prevalence divided by 100) is >0.175 if the prevalence is ≤50%, 3) the RSE of the negative of the natural logarithm of (1-p) is >0.175 if the prevalence is >50%, 4) the actual sample size is <100, or 5) the effective sample size (defined as the actual sample size divided by the design effect) is <68.

^¶^ Persons of Hispanic origin can be of any race.

** Includes persons with a general education diploma.

^††^ County type estimates are based on the 2013 Rural-Urban Continuum Codes. https://www.ers.usda.gov/data-products/rural-urban-continuum-codes.aspx

^§§^ Area with a population of ≥1 million persons.

^¶¶^ Area with a population of <1 million persons.

*** Area that is outside of a metropolitan statistical area.

**^†††^** Methodological changes that occurred in 2015 had minimal effects on estimates provided in this report.

^§§§^ Estimates are based on a definition of the poverty level that incorporates information on family income, size, and composition and is calculated as a percentage of the U.S. Census Bureau's poverty thresholds. Respondents aged 18–22 years who were living in a college dormitory were excluded.

^¶¶¶^ Respondents could indicate multiple types of health insurance; thus, these response categories are not mutually exclusive.

**** Persons aged ≤19 years are eligible for this plan.

**^††††^** Defined as having Medicare, CHAMPUS, TRICARE, CHAMPVA, Veterans Administration, military health care, or any other type of health insurance.

^§§§§^ *Northeast*: Connecticut, Maine, Massachusetts, New Hampshire, New Jersey, New York, Pennsylvania, Rhode Island, and Vermont. *Midwest*: Illinois, Indiana, Iowa, Kansas, Michigan, Minnesota, Missouri, Nebraska, North Dakota, Ohio, South Dakota, and Wisconsin. *South*: Alabama, Arkansas, Delaware, District of Columbia, Florida, Georgia, Kentucky, Louisiana, Maryland, Mississippi, North Carolina, Oklahoma, South Carolina, Tennessee, Texas, Virginia, and West Virginia. *West*: Alaska, Arizona, California, Colorado, Hawaii, Idaho, Montana, Nevada, New Mexico, Oregon, Utah, Washington, and Wyoming.

^¶¶¶¶^ Totals exclude persons with missing or unknown race and ethnicity. Totals might vary due to rounding**.**

## Discussion

The findings in this report indicate that the prevalence of suicidal thoughts, plans, and attempts varies by selected sociodemographic characteristics and across states and regions. The study showed that the prevalence of suicidal thoughts, plans, and attempts varies by sex. Females were more likely than males to have had suicidal thoughts and to make plans to kill themselves and attempt suicide. In addition, females were more likely than males to have had suicidal attempts that required medical attention and hospitalization. This study also showed that the prevalence of most suicidal thoughts and behaviors varied by race and ethnicity. Non-Hispanic AI/AN adults had the highest prevalence of having made any suicide plans compared with any other single racial or ethnic group. Non-Hispanic persons of two or more races had higher prevalence rates of having had suicidal thoughts when compared with other racial or ethnic groups. Non-Hispanic White persons were more likely to have had suicidal thoughts than Black, Asian, NH/OPI, and Hispanic persons, and more likely to have made suicide plans than Black, Asian, and Hispanic adults. Non-Hispanic White and Non-Hispanic Asian adults were equally less likely to attempt suicide than Black, AI/AN, and NH/OPI adults, persons of two or more races, and Hispanic adults.

Variations in the prevalence of suicidal thoughts and behavior among demographic groups can be attributed to several factors, including economic (e.g., poverty, unemployment, low educational attainment, deprivation, access to health care, and being unhoused); safety threats such as conflicts (e.g., wars and civil strife) and disasters (e.g., natural or human-made); the co-occurrence of major physical diseases such as AIDS, diabetes, or cancer; and the familial, cultural, and social environment (e.g., genetic predisposition to disease, coping skills, accumulation of stressful life events [such as loss of a job, marriage, death of a loved one, or change of residence], discrimination, stigma against help-seeking, or mistrust of health care providers) (*32*).

The prevalence of suicidal thoughts and behaviors varied across states and by region. These data might be helpful in developing, improving, and evaluating state, regional, and local policies, programs, and practices. NSDUH can also be used to compare data from the state and large metropolitan surveys because they share similar sample designs, questionnaires, and both data collection and processing procedures. This study does not describe patterns in large metropolitan areas; however, previous studies did show variations in the prevalence of suicidal thoughts and behavior across metropolitan statistical areas (*33*,*34*). The variations might reflect differences in state policies, regional access to services, availability of effective community prevention programs, prevailing behavioral and social norms, or differences in the demographic characteristics of the population.

Organizations have used NSDUH data on suicide-related items in various reports and publications to stimulate support for and improvements in public health initiatives, including the 2012 National Strategy for Suicide Prevention (*2*), the response to the 2010 Deep Water Horizon oil spill in the Gulf of Mexico (*35*,*36*), and the national Garrett L. Smith Memorial Act suicide prevention program (*37*). At the state and local level, health and education agencies and nongovernmental organizations use NSDUH data to improve health-related policies, programs, and practices. State health departments’ continued use of these data to describe the burden of suicidal thoughts and behavior might increase awareness of the issue and support wider adoption of prevention efforts (*38*). State-specific NSDUH data can also help to describe the scope of the issue, identify areas of need within a specific county (*39*), and increase vigilance for preventing suicidal thoughts and behavior (*40*).

Suicidal behavior is preventable and multiple strategies have shown success in decreasing fatal and nonfatal suicidal behaviors or key risk factors for suicide. CDC’s technical package for preventing suicide (*24*) describes seven strategies that are based on the best available evidence, including strengthening economic supports, strengthening access and delivery of suicide care, creating protective environments, promoting connectedness, teaching coping and problem-solving skills, identifying and supporting persons at risk, and lessening harms and preventing future risk.

## Limitations

The findings in this report are subject to at least four limitations. First, because of low precision, certain estimates were suppressed after stratification by state and sociodemographic characteristics, which limited the ability to examine more age groups and certain racial and ethnic populations. However, much of the data presented in this report are comparable with those of previous studies examining similar types of thoughts and behaviors (*41*). Second, estimates presented in this report using 2015–2019 NSDUH data are generalizable only to the civilian, noninstitutionalized U.S. population aged ≥18 years. Although this population includes most of the total U.S. population aged ≥18 years, it excludes active-duty military personnel, persons living in institutional group quarters (e.g., correctional facilities and residential drug treatment centers), and unhoused persons not living in shelters on the survey date. These populations have a higher prevalence of some suicidal thoughts and behaviors than the general U.S. population, and thus this report likely underestimates the actual prevalence of suicidal thoughts and behaviors among adults in the United States (*42*). Third, NSDUH data are based on self-reports and not observations of actual incidents in the case of suicide attempts, and the extent of underreporting or overreporting of behaviors was not assessed. Similar survey questions that were administered among high school students demonstrated good test-retest reliability (*43*). However, no reliability or validity data were obtained on the measures of suicidal thoughts, plans, and attempts among adults. Finally, because of the cross-sectional nature of the survey, the results cannot be used to infer temporal or causal associations between the variables examined and suicidal behavior.

## Future Directions

The provisional number of suicides and the age-adjusted suicide rate in 2020 were 3% lower than in 2019 (*44*). It will be important to continue to evaluate future years of NSDUH data to determine if or how this might translate to changes in prevalence of suicidal thoughts and behaviors in adults. Because suicidal behavior is affected by a combination of factors (e.g., individual-, family-, community-, and societal-level), prevention efforts are more likely to succeed if they combine multiple strategies that work together to prevent the behavior (*45*). These comprehensive strategies often include multiple components including relevant timely data such as that collected by NSDUH (*24*,*46*,*47*). Additional data sources are either being conceptualized or already under development. One example is real-time hospital emergency department data from the Emergency Department Surveillance of Nonfatal Suicide-Related Outcomes (*48*,*49*). Other sources include local data that would be useful in planning and implementing local prevention programs. Successfully addressing suicidal behavior requires assessing the best available evidence, assuring adequate resources, ongoing study of the issue to identify changes, and adopting new strategies when appropriate (*50*).

## Conclusion

NSDUH is an annual source of high-quality data at the national, state, and large metropolitan levels for monitoring health behaviors that contribute to the leading causes of mortality and morbidity among adults in the United States. These data enable researchers to estimate the prevalence of suicidal thoughts and behaviors among adults with different sociodemographic characteristics. NSDUH data are an important tool for planning, implementing, and evaluating public health policies, programs, and practices to reduce the overall burden of suicide, especially at the state and regional levels. These data also allow for future analyses to investigate the associations between other health behaviors (e.g., alcohol and other drug use) and suicidal thoughts and behaviors and for assessing trends among subgroups of adults (e.g., by sex or race/ethnicity) at the state and large metropolitan levels. These analyses support more recent efforts in public health to build and sustain the momentum of a comprehensive approach to suicide prevention.

## Footnotes

* 45 C.F.R. part 46; 21 C.F.R. part 56.

† The questionnaires for the study period are available at https://www.samhsa.gov/data/data-we-collect/nsduh-national-survey-drug-use-and-health.

§ Persons of Hispanic origin can be of any race.

¶ *Northeast*: Connecticut, Maine, Massachusetts, New Hampshire, New Jersey, New York, Pennsylvania, Rhode Island, and Vermont. *Midwest*: Illinois, Indiana, Iowa, Kansas, Michigan, Minnesota, Missouri, Nebraska, North Dakota, Ohio, South Dakota, and Wisconsin. *South*: Alabama, Arkansas, Delaware, DC, Florida, Georgia, Kentucky, Louisiana, Maryland, Mississippi, North Carolina, Oklahoma, South Carolina, Tennessee, Texas, Virginia, and West Virginia. *West*: Alaska, Arizona, California, Colorado, Hawaii, Idaho, Montana, Nevada, New Mexico, Oregon, Utah, Washington, and Wyoming.

## References

[1] Hedegaard H, Curtin SC, Warner M. Suicide mortality in the United States, 1999–2017. NCHS Data Brief, no 330. Atlanta, GA: US Department of Health and Human Services, CDC, National Center for Health Statistics; 2018. https://www.cdc.gov/nchs/products/databriefs/db330.htm

[2] US Department of Health and Human Services, Office of the Surgeon General, National Action Alliance for Suicide Prevention. 2012 national strategy for suicide prevention: goals and objectives for action. Washington, DC: US Department of Health and Human Services; 2012. https://www.ncbi.nlm.nih.gov/books/NBK109917/23136686

[3] Crosby AE, Ortega L, Melanson C. Self-directed violence surveillance: uniform definitions and recommended data elements, version 1.0. Atlanta, GA: US Department of Health and Human Services, CDC; 2011. https://www.cdc.gov/violenceprevention/pdf/self-directed-violence-a.pdf

[4] CDC. Web-Based Injury Statistics Query and Reporting System. Atlanta, GA: US Department of Health and Human Services, CDC; 2021. https://www.cdc.gov/injury/wisqars/index.html

[5] Stone DM, Simon TR, Fowler KA, Trends in state suicide rates—United States, 1999–2016 and circumstances contributing to suicide—27 states, 2015. MMWR Morb Mortal Wkly Rep 2018;67:617–24. 10.15585/mmwr.mm6722a1 29879094 PMC5991813

[6] Agency for Healthcare Research and Quality. Healthcare Cost and Utilization Project. HCUP NIS database documentation. Rockville, MD: US Department of Health and Human Services, Agency for Healthcare Research and Quality; 2018. https//hcup-us.ahrq.gov/db/nation/nis/nisdbdocumentation.jsp

[7] Substance Abuse and Mental Health Services Administration. Results from the 2017 National Survey on Drug Use and Health: detailed tables. Rockville, MD: US Department of Health and Human Services, Substance Abuse and Mental Health Services Administration; 2017. https://www.samhsa.gov/data/report/2017-nsduh-detailed-tables

[8] Cerel J, Brown MM, Maple M, How many people are exposed to suicide? Not six. Suicide Life Threat Behav 2019;49:529–34. 10.1111/sltb.12450 29512876

[9] Berman AL. Estimating the population of survivors of suicide: seeking an evidence base. Suicide Life Threat Behav 2011;41:110–6. 10.1111/j.1943-278X.2010.00009.x 21309829

[10] Satcher D. Bringing the public health approach to the problem of suicide. Suicide Life Threat Behav 1998;28:325–7. 9894300

[11] Rockett IRH. Counting suicides and making suicide count as a public health problem. Crisis 2010;31:227–30. 10.1027/0227-5910/a000071 21134841

[12] Blair JM, Fowler KA, Jack SPD, Crosby AE. The National Violent Death Reporting System: overview and future directions. Inj Prev 2016;22(Suppl 1):6–11. 10.1136/injuryprev-2015-041819 26718549 PMC6049659

[13] Ivey-Stephenson AZ, Blair JM, Crosby AE. Efforts and opportunities to understand women’s mortality due to suicide and homicide using the National Violent Death Reporting System. J Womens Health (Larchmt) 2018;27:1073–81. 10.1089/jwh.2018.7320 30192184 PMC6822102

[14] Crosby AE, Ikeda R, Hedegaard H, ; Data and Surveillance Task Force of the National Action Alliance for Suicide Prevention. Improving national data systems for surveillance of suicide-related events. Am J Prev Med 2014;47(Suppl 2):S122–9. 10.1016/j.amepre.2014.05.026 25145729 PMC4959537

[15] Agency for Healthcare Research and Quality. Healthcare Cost and Utilization Project. Rockville, MD: US Department of Health and Human Services, Agency for Healthcare Research and Quality; 2021. https://www.ahrq.gov/data/hcup/index.html

[16] Sethi D, Habibula S, McGee K, , eds. Guidelines for conducting community surveys on injuries and violence. Geneva, Switzerland: World Health Organization; 2004. https://apps.who.int/iris/handle/10665/42975 10.1080/156609704/233/327505 15903167

[17] Ha JF, Longnecker N. Doctor-patient communication: a review. Ochsner J 2010;10:38–43. 21603354 PMC3096184

[18] Lavallee DC, Chenok KE, Love RM, Incorporating patient-reported outcomes into health care to engage patients and enhance care. Health Aff (Millwood) 2016;35:575–82. 10.1377/hlthaff.2015.1362 27044954

[19] Nock MK, Borges G, Bromet EJ, Cha CB, Kessler RC, Lee S. Suicide and suicidal behavior. Epidemiol Rev 2008;30:133–54. 10.1093/epirev/mxn002 18653727 PMC2576496

[20] Hanlon C, Rosenthal J, Hinkle L. State documentation of racial and ethnic health disparities to inform strategic action. Rockville, MD: US Department of Health and Human Services, Agency for Healthcare Research and Quality; 2011. https://www.hcup-us.ahrq.gov/reports/race/stand_alone_r_e_rpt.jsp

[21] Ivey-Stephenson AZ, Crosby AE, Jack SPD, Haileyesus T, Kresnow-Sedacca MJ. Suicide trends among and within urbanization levels by sex, race/ethnicity, age group, and mechanism of death—United States, 2001–2015. MMWR Surveill Summ 2017;66(No. SS-18):1–16. 10.15585/mmwr.ss6618a1 28981481 PMC5829833

[22] Crosby AE, Han B, Ortega LAG, Parks SE, Gfroerer J. Suicidal thoughts and behaviors among adults aged ≥18 years—United States, 2008–2009. MMWR Surveill Summ 2011;60(No. SS-13):1–22. 22012169

[23] Ribeiro JD, Franklin JC, Fox KR, Self-injurious thoughts and behaviors as risk factors for future suicide ideation, attempts, and death: a meta-analysis of longitudinal studies. Psychol Med 2016;46:225–36. 10.1017/S0033291715001804 26370729 PMC4774896

[24] Stone DM, Holland KM, Bartholow B, Crosby AE, Davis S, Wilkins N. Preventing suicide: a technical package of policies, programs, and practices. Atlanta, GA: US Department of Health and Human Services, CDC, National Center for Injury Prevention and Control; 2017. https://www.cdc.gov/violenceprevention/pdf/suicideTechnicalPackage.pdf

[25] Substance Abuse and Mental Health Services Administration. Key substance use and mental health indicators in the United States: results from the 2015 National Survey on Drug Use and Health. HHS Publication No. SMA 16–4984, NSDUH Series H-51. Rockville, MD: US Department of Health and Human Services, Substance Abuse and Mental Health Services Administration; https://www.samhsa.gov/data

[26] Substance Abuse and Mental Health Services Administration. Key substance use and mental health indicators in the United States: results from the 2016 National Survey on Drug Use and Health. HHS Publication No. SMA 17–5044, NSDUH Series H-52. Rockville, MD: US Department of Health and Human Services, Substance Abuse and Mental Health Services Administration; 2017. https://www.samhsa.gov/data

[27] Substance Abuse and Mental Health Services Administration. Key substance use and mental health indicators in the United States: Results from the 2017 National Survey on Drug Use and Health. HHS Publication No. SMA 18–5068, NSDUH Series H-53. Rockville, MD: US Department of Health and Human Services, Substance Abuse and Mental Health Services Administration; 2018. https://www.samhsa.gov/data

[28] Substance Abuse and Mental Health Services Administration. Key substance use and mental health indicators in the United States: results from the 2018 National Survey on Drug Use and Health. HHS Publication No. PEP19–5068, NSDUH Series H-54. Rockville, MD: US Department of Health and Human Services, Substance Abuse and Mental Health Services Administration; 2019. https://www.samhsa.gov/data/

[29] Substance Abuse and Mental Health Services Administration. Key substance use and mental health indicators in the United States: Results from the 2019 National Survey on Drug Use and Health (HHS Publication No. PEP20–07–01–001, NSDUH Series H-55). Rockville, MD: US Department of Health and Human Services, Substance Abuse and Mental Health Services Administration; 2020. https://www.samhsa.gov/data

[30] US Department of Agriculture, Economic Research Service. Measuring rurality: 2013 county typology codes. Washington, DC: US Department of Agriculture, Economic Research Service; 2013. https://www.ers.usda.gov/data-products/rural-urban-continuum-codes.aspx

[31] Substance Abuse and Mental Health Services Administration. 2019 National Survey on Drug Use and Health: methodological summary and definitions. Rockville, MD: US Department of Health and Human Services, Substance Abuse and Mental Health Services Administration; 2020. https://www.samhsa.gov/data/

[32] Lorant V, Kunst AE, Huisman M, Costa G, Mackenbach J. EU Working Group on Socio-Economic Inequalities in Health. Socio-economic inequalities in suicide: a European comparative study. Br J Psychiatry 2005;187:49–54. 10.1192/bjp.187.1.49 15994571

[33] Substance Abuse and Mental Health Services Administration. The NSDUH report: suicidal thoughts and behavior in 33 metropolitan statistical areas: 2008 to 2010. Rockville, MD: US Department of Health and Human Services, Substance Abuse and Mental Health Services Administration; 2012. https://www.samhsa.gov/data/sites/default/files/NSDUH119/NSDUH119/SR119SuicideByMSA2012.pdf

[34] Park-Lee E, Hedden SL, Lipari RN. The NSDUH report: suicidal thoughts and behavior in 33 metropolitan statistical areas update: 2013 to 2015. Rockville, MD: US Department of Health and Human Services, Substance Abuse and Mental Health Services Administration; 2017. https://www.samhsa.gov/data/sites/default/files/report_3452/ShortReport-3452.html 29659227

[35] Gould DW, Teich JL, Pemberton MR, Pierannunzi C, Larson S. Behavioral health in the gulf coast region following the Deepwater Horizon oil spill: findings from two federal surveys. J Behav Health Serv Res 2015;42:6–22. 10.1007/s11414-014-9441-8 25339594 PMC4700882

[36] Substance Abuse and Mental Health Services Administration. CDC. Behavioral health in the gulf coast region following the Deepwater Horizon oil spill. HHS Publication No. SMA 13–4737. Rockville, MD: US Department of Health and Human Services, Substance Abuse and Mental Health Services Administration, CDC; 2013. https://www.samhsa.gov/data/sites/default/files/NSDUH-GSPS-GulfCoast-Apps-2012/NSDUH-GSPS-GulfCoast-2012.pdf

[37] Godoy Garraza L, Walrath C, Goldston DB, Reid H, McKeon R. Effect of the Garrett Lee Smith Memorial Suicide Prevention Program on suicide attempts among youths. JAMA Psychiatry 2015;72:1143–9. 10.1001/jamapsychiatry.2015.1933 26465226

[38] Substance Abuse and Mental Health Services Administration. Behavioral health barometer: Missouri, 2013. Rockville, MD: US Department of Health and Human Services, Substance Abuse and Mental Health Services Administration; 2013. https://www.samhsa.gov/data/sites/default/files/State_BHBarometers_2014_1/BHBarometer-MO.pdf

[39] Middlesex County Substance Abuse Action Council. Suicide prevention: what is the scope of suicidal behavior in Middlesex County? Middletown, CT: Middlesex County Substance Abuse Action Council; 2021. http://mcsaac.org/suicide-prevention

[40] Lipari RN, Hughes A, Williams M. State estimates of past year serious thoughts of suicide among young adults: 2013 and 2014. Rockville, MD: Substance Abuse and Mental Health Services Administration; 2016. https://www.samhsa.gov/data/sites/default/files/report_2387/ShortReport-2387.pdf 27854411

[41] Miller GK, Piscopo KD, Batts K, Measurement of suicidal thoughts, behaviors, and related health outcomes in the United States: comparison of NSDUH estimates with other data sources. Rockville, MD: US Department of Health and Human Services, Substance Abuse and Mental Health Services Administration; 2015. https://www.samhsa.gov/data/sites/default/files/NSDUH-DR-N20Suicide-2015/NSDUH-DR-N20Suicide-2015.htm

[42] Mumola C, Noonan M. Deaths in custody statistical tables. Washington, DC: US Department of Justice, Office of Justice Programs, Bureau of Justice Statistics; 2008. https://bjs.ojp.gov/library/publications/deaths-custody-state-prison-deaths-2001-2007-statistical-tables

[43] Brener ND, Kann L, McManus T, Kinchen SA, Sundberg EC, Ross JG. Reliability of the 1999 youth risk behavior survey questionnaire. J Adolesc Health 2002;31:336–42. 10.1016/S1054-139X(02)00339-7 12359379

[44] Curtin SC, Hedegaard H, Ahmad FB. Provisional numbers and rates of suicide by month and demographic characteristics: United States, 2020. Vital Statistics Rapid Release; no 16. Hyattsville, MD: US Department of Health and Human Services, CDC, National Center for Health Statistics; 2021. https://www.cdc.gov/nchs/data/vsrr/VSRR016.pdf

[45] World Health Organization. Preventing suicide: a global imperative. Geneva, Switzerland: World Health Organization; 2014. https://www.who.int/publications/i/item/9789241564779

[46] Caine ED, Reed J, Hindman J, Quinlan K. Comprehensive, integrated approaches to suicide prevention: practical guidance. Inj Prev 2018;24(Suppl 1):i38–45. 10.1136/injuryprev-2017-042366 29263088

[47] Office of the White House Press Secretary. Reducing military and veteran suicide: advancing a comprehensive, cross-sector, evidence-informed public health strategy. https://www.whitehouse.gov/wp-content/uploads/2021/11/Military-and-Veteran-Suicide-Prevention-Strategy.pdf

[48] US Department of Health and Human Services. The Surgeon General’s call to action to implement the national strategy for suicide prevention: a report of the U.S. Surgeon General and the National Action Alliance for Suicide Prevention. Washington, DC: US Public Health Service; 2021. https://www.hhs.gov/sites/default/files/sprc-call-to-action.pdf

[49] CDC. Emergency department surveillance of nonfatal suicide-related outcomes. Atlanta, GA: US Department of Health and Human Services, CDC; 2021. https://www.cdc.gov/suicide/programs/ed-snsro

[50] National Action Alliance for Suicide Prevention. Transforming communities: key elements for the implementation of comprehensive community-based suicide prevention. Washington, DC: Education Development Center; 2017. https://theactionalliance.org/sites/default/files/transformingcommunitiespaper.pdf

